# Experimental Study on Bond Performance of Carbon- and Glass-Fiber Reinforced Polymer (CFRP/GFRP) Bars and Steel Strands to Concrete

**DOI:** 10.3390/ma14051268

**Published:** 2021-03-07

**Authors:** Jun Zhao, Xin Luo, Zike Wang, Shuaikai Feng, Xinglong Gong, Eskinder Desta Shumuye

**Affiliations:** 1 School of Mechanics and Safety Engineering, Zhengzhou University, Zhengzhou 450001, China; zhaoj@zzu.edu.cn (J.Z.); luoxin199505@163.com (X.L.); gongxl@ustc.edu.cn (X.G.); 2 School of Civil Engineering, Zhengzhou University, Zhengzhou 450001, China; ffengsk@163.com (S.F.); eskdes@gs.zzu.edu.cn (E.D.S.); 3 CAS Key Laboratory of Mechanical Behavior and Design of Materials, Department of Modern Mechanics, University of Science and Technology of China, Hefei 230027, China

**Keywords:** CFRP bars, GFRP bars, steel strands, bond strength, residual bond strength, bond strength retention rate

## Abstract

FRP bars and steel strands are widely used in civil engineering. In this study, three different types of high-strength reinforcement materials, carbon fiber reinforced polymer (CFRP) bar, glass fiber reinforced polymer (GFRP) bar, and steel strand, were investigated for their interfacial bond performance with concrete. A total of 90 sets of specimens were conducted to analyze the effects of various parameters such as the diameter of reinforcement, bond length, the grade of concrete and stirrup on the bond strength and residual bond strength. The results show that CFRP bars possess a higher bond strength retention rate than steel bars in the residual section. In addition, with the increase in bond length and diameter of the CFRP bar, the residual bond strength decreases, and the bond strength retention rate decreases. Furthermore, the bond strength retention rate of GFRP bars was found to be higher than that of CFRP bars. With the increase in grade of concrete, the bond strength and residual bond strength between GFRP bars and concrete increases, but the bond strength retention rate decreases. With an increase in bond length and diameter of the GFRP bar, the bond strength starts to decrease. Further, stirrup can also increase the bond strength and reduce the slip at the free end of GFRP bars. Moreover, the bond strength retention rate of the steel strand was found to be lower than CFRP and GFRP bar.

## 1. Introduction

So far, steel bars have been mostly used in traditional reinforced concrete structures. During its service period, it is inevitable to face various environmental exposure such as (humidity, water, acid rain, deicing salt, and seawater), and hence the corrosion of steel bar in reinforced concrete structures becomes a serious problem [1,2,3]. In addition, the interface between steel bar and concrete can be altered by the corrosion process of steel bar, resulting in a reduction in the bond strength between steel bar and concrete, further leading to a shortened service life of the reinforced concrete structure. In the United States, Canada, and European countries, this creates a huge economic burden during regular maintenance, repair, and restoration [4,5] of reinforced concrete structures, which has prompted an urgent need for alternative reinforcement measures. One of the proposed solutions for these problems is the use of fiber-reinforced polymer (FRP) bars with excellent corrosion resistance to replace steel bars as the novel internal reinforcements for concrete structures [6,7,8,9,10].

FRP bars are composed of reinforcing fibers, resin matrix, and fiber–resin interface [11]. The fibers play a reinforcing role and mainly determine the mechanical properties of FRP [12,13,14], such as stiffness, modulus, and strength. The resin mainly plays a shaping role and is used to fix and protect the fibers, commonly includes unsaturated polyester, epoxy resin, and vinyl resin, etc. The fiber–resin interface, as a transition area, mainly undertakes stress transfer between fiber and resin to ensure that the fibers and resin can work together as a whole. FRP bars for civil engineering are mainly produced by the pultrusion process, and according to the type of fiber, the traditional FRP bars mainly include glass FRP (GFRP), carbon FRP (CFRP), and aramid FRP (AFRP). Due to its excellent mechanical properties, FRP-reinforced concrete has been successfully used in many structural fields such as water conservancy projects, building construction, and highways [15,16,17].

Many scholars have investigated the bond behavior between FRP bars and concrete, and the key influence factors on the bond properties are the diameter of bars, bond length, grade of the concrete, stirrup, etc. [18,19]. The surface morphology of FRP bars also affects the bond strength [20,21]. Furthermore, many researchers also investigated the bond performance between FRP bars and concrete under various extreme conditions, such as aggressive water/alkali environments, seawater conditions, elevated temperatures, etc. [22,23,24,25,26,27,28].

Meanwhile, steel strand has also attracted the attention of civil engineering researchers due to its ultra-high strength. Steel strand is a steel product composed of multiple steel wires stranded together, and the surface of carbon steel can be coated with protection layers such as galvanized, zinc-aluminum alloy, aluminum clad, copper plating, and epoxy. However, only limited experimental results were reported on the bond performance between steel strands and concrete. In the 1977, Salmons [29] investigated the effect of several factors on the bond strength between steel strand and concrete, such as the diameter of steel strand, grade of concrete, and bond length. Furthermore, Gustavson [30] investigated the effect of three influence factors, including the grade of concrete, density of concrete, and shape of the steel strand profile. Recently, Martí-Vargas [31] also studied the effect of concrete composition with different cement contents and water/cement ratios on the bond behavior of seven-wire prestressing strands to concrete. 

Most previous studies investigated the bond performance between FRP bars/steel strands and concrete [32,33,34,35,36,37], and these studies mainly focused on bond strength (i.e., maximum bond stress) and the ascending and descending sections of the bond-slip curve. However, few studies focused on the residual section of bond-slip curves. In fact, the bond performance in the residual section (i.e., post-descending stage) of the bond–slip curve also plays an important role in the design of reinforced concrete structures, especially during the analysis and evaluation of the recoverability of structures after the earthquake. Therefore, this paper focuses on the analysis of the whole complete stage, including ascending, descending, and residual sections of bond–slip curves of FRP bars/steel strands to concrete.

In this study, CFRP/GFRP bars and steel strands were tested and investigated for their interfacial bond–slip performance with concrete, while steel bars were adopted as the control specimens. The novelty of this paper is that firstly systematically investigated the residual section of bond–slip curves between three kinds of high-strength reinforcements (CFRP bars, GFRP bars, and steel strands) and concrete. A total of 90 sets of pull-out specimens were tested to analyze the effects of reinforcement diameter, bond length, concrete grade, and stirrup on bond strength, residual bond strength, and bond–slip curve. The results of this study will widen the understanding of the bond performance of FRP bars and steel strands to concrete, and promote the application of FRP bar during the design of the concrete structure in earthquake-prone areas.

## 2. Materials and Method

### 2.1. FRP Bars and Steel Strands

In this study, CFRP bars with nominal diameters of 10 mm and 12 mm, GFRP bars with nominal diameters of 12 mm and 20 mm, and seven-wire steel strands with diameters of 12.7 mm and 15.2 mm were adopted for each experimental works. CFRP bars, purchased from Shenzhen Oceanpower New Material Technology Co., Ltd. (Shenzhen, China), were made of Zhongfu Shenying SYT49S carbon fiber (close to the level of Toray T700) and epoxy resin through pultrusion. GFRP bars, purchased from Nanjing Fenghui Composite Material Co., Ltd. (Nanjing, China), were made of E-glass fiber and unsaturated polyester resin by pultrusion. According to ASTM D 7205/D7205 M-06 [38], the tensile strength and modulus of elasticity were tested, as shown in Table 1. Meanwhile, HRB335 steel bars with a nominal diameter of 12 mm were used as a control group.

**Figure 1:**
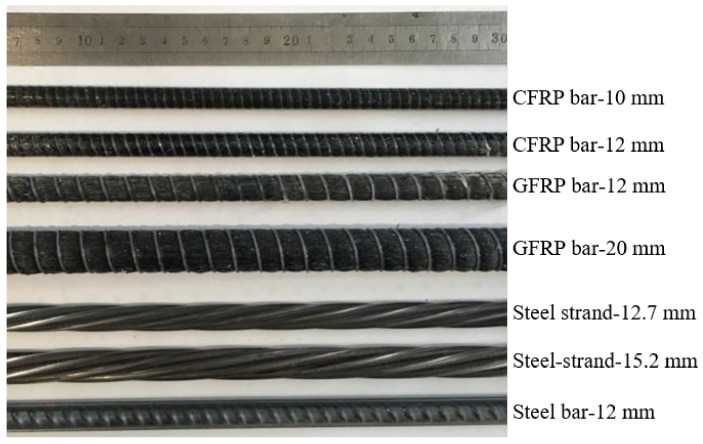
Surface morphologies of reinforcements.

The detailed surface morphologies of CFRP, GFRP, and steel bars and steel strands are shown in Figure 1, respectively. It can be found that the surface of CFRP and GFRP bars were all helically wrapped with a polyester fiber bundle during pultrusion, forming the ribs on their surface to improve the bonding between FRP bars and concrete. The measured distance between ribs for CFRP bars with diameters of 10 mm and 12 mm were about 4.8 mm and 5.5 mm, respectively. The measured distance between ribs for GFRP bars with diameters of 12 mm and 20 mm were about 10.8 mm and 11.9 mm, respectively. The measured depth rib on the surface of CFRP bars and GFRP bars were about 0.1 mm and 1.0 mm, respectively.

### 2.2. Concrete

Three kinds of concrete with a strength grade of C30, C40, and C50 were prepared according to the mix proportion shown in Table 2, and the raw materials include fine aggregate (river sand) with a particle size of 0.25–0.5 mm, coarse aggregate (crushed gravel) with a particle size range of 5–20 mm. Additionally, tap water and polycarboxylate (Zhengzhou Jetbon New Material Co., Ltd., Zhengzhou, China) as a water reducer was adopted. The compressive strength was determined by casting three cubes of dimension 150 mm × 150 mm × 150 mm, under standard curing conditions (20 °C; 95% relative humidity). The measured compressive strengths of concrete specimens cured for 28 days were shown in Table 2. 

### 2.3. Pull-Out Tests

**Figure 2:**
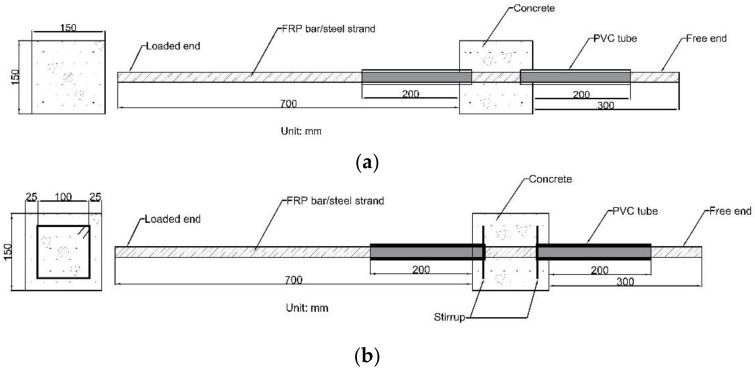
Schematic diagram of a pull-out specimen (**a**) without stirrup and (**b**) with the stirrup. (Unit: mm).

The bond properties of various reinforcements to concrete were tested using the central pull-out test method. As shown in Figure 2, the pull-out specimens with (150 mm × 150 mm × 150 mm) concrete block, with reinforcement extending 300 mm at the free end and 700 mm at the loaded end were designed. For the pull-out specimen with stirrups, two sets of steel stirrups with a size of 100 mm × 100 mm and 25 mm concrete cover were additionally added. Based on Losberg’s recommendation [39], the bonding zone of reinforcement to concrete was placed in the middle part of the concrete block, and the debonding was placed at both ends. PVC tubes were used as the debonding devices during the casting process of pull-out specimens.

**Figure 3:**
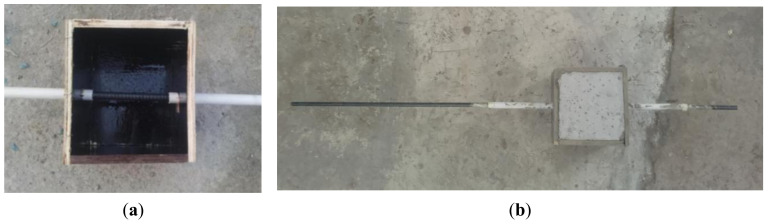
Photograph of the pull-out specimen: (**a**) mold and (**b**) cured specimen block.

As shown in Figure 3a, before casting the pull-out specimens, the PVC tubes were first inserted into the wooden mold, and the PVC tubes were fixed according to the bond length. Then, the reinforcement was inserted into the PVC tubes, and the gaps between reinforcement and PVC were sealed to avoid the penetration of concrete mortar/paste into the debonding zones during the casting process. After casting, all specimens were covered with a polythene sheet to prevent evaporation of water from the unhardened concrete until demolding for 48 h and stored in the standard curing condition until testing (28 days). The pull-out specimen before demolding is shown in Figure 3b.

**Figure 4:**
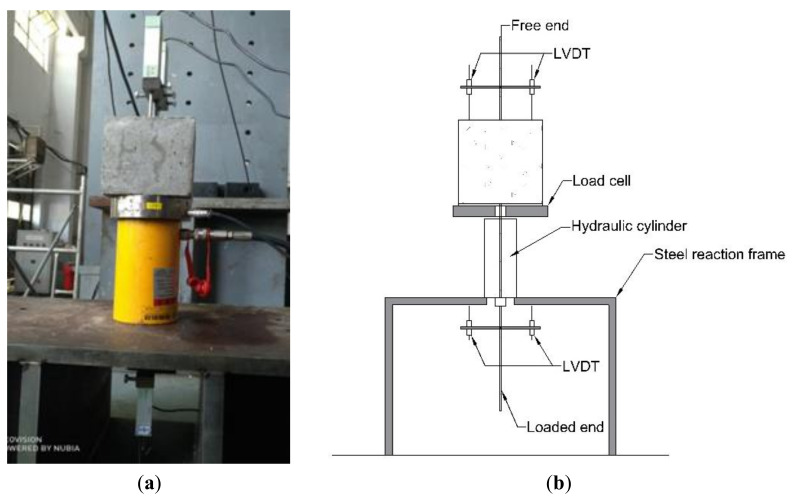
Test device: (**a**) photograph and (**b**) schematic diagram.

The pull-out test was conducted using a set of devices developed by the laboratory. As shown in Figure 4, a 200 kN hydraulic cylinder is first inverted on the steel reaction frame with a hole, and a load cell (Jinan Taiqin Electric Co, Ltd., Jinan, China) is placed on the upper of the cylinder. Then, the pull-out specimen was placed on the devices with the concrete block placed on the upper of the cell, and the reinforcement passed through the load cell, hydraulic cylinder, and steel reaction frame. Finally, the lower end of reinforcement, which is the loading end, is fixed with the anchor, and the hydraulic cylinder is manually loaded to push the anchor downward so that the reinforcement is subjected to the downward pulling force until the end of the test. Two linear variable differential transformers (LVDTs, Liyang Instrument Factory, Liyang, China) were placed at the free end and loaded end to measure the slip and during the pull-out test, and the load cell and LVDTs were connected to a static strain acquisition instrument to collect the force and displacement data. The slip result of the specimen equals the average of LVDT displacements at the free or loaded end. The pull-out test was manually loaded with a loading rate of about 0.2 mm/min.

### 2.4. Definition of Specimen Label

In this study, a total of 30 groups of pull-out specimens were tested, which includes seven groups of CFRP, 10 groups of GFRP, 11 groups of steel strands, and two groups of steel bars. Three specimens were repeated in each group. Among these tests, the main influencing parameters include the type of reinforcement, grade of concrete, diameter and bond strength of reinforcement, and stirrup were considered.

For simplicity, the definition of pull-out specimen label was as follows: the specimen label was based on “reinforcement type, concrete grade-reinforcement diameter-bond length-stirrup.” For the reinforcement type, “C” represents CFRP bar, “G” represents GFRP bar, “SB” represents steel bar, and “SS” represents steel strand; the first label represents the strength grade of concrete, e.g., “30” and “50” represent the concrete with grades of C30 and C50, respectively; the second label stands for the reinforcement diameter, e.g., “10” and “12.7” represents the diameter of 10 mm and 12.7 mm, respectively; the third label stands for the bond length, e.g., “5” and “7.5” represent the bond length of 5 times and 7.5 times diameter of reinforcement, respectively; the last letter stands for whether the stirrups were set, as in “S” represents the stirrups. It is noted that for the pull-out specimens without stirrups, the last letter “S” is missing in its label.

For example, “C30-10-7.5-S” stands for the pull-out specimen with CFRP bar, concrete of grade of C30, diameter of 10 mm, bond length of 7.5 times the diameter of CFRP bar, and stirrups. “SB30-12-5” stands for the pull-out specimen with a steel bar, concrete of grade of C30, a diameter of 12 mm, the bond length of five times the diameter of steel bar, and without stirrups.

## 3. Test Results

### Typical Bond–Slip Curves and Failure Modes

**Figure 5:**
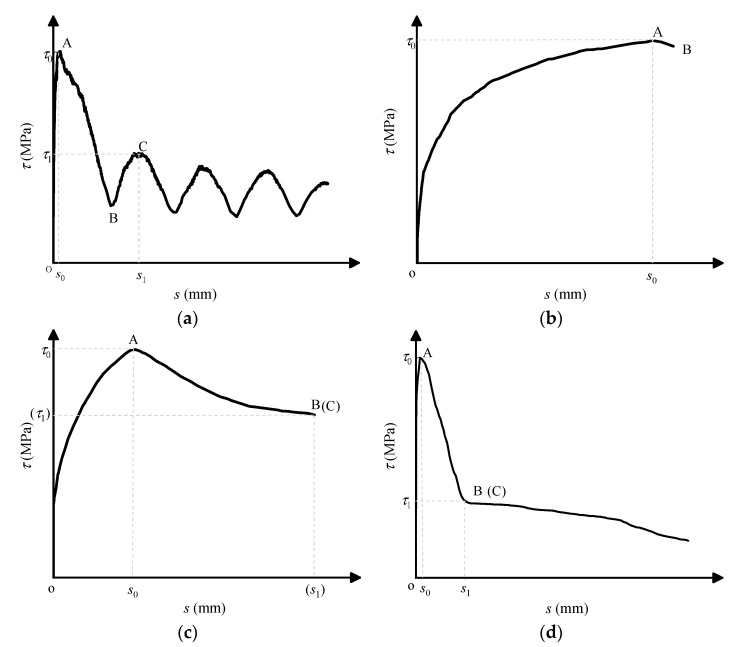
Typical Bond–slip (*τ*–*s*) curves of pull-out specimens: (**a**) fiber-reinforced polymer (FRP) bars with pull-out failure; (**b**) FRP bars with splitting failure; (**c**) steel strands with pull-out failure; and (**d**) steel bars with pull-out failure.

**Figure 6:**
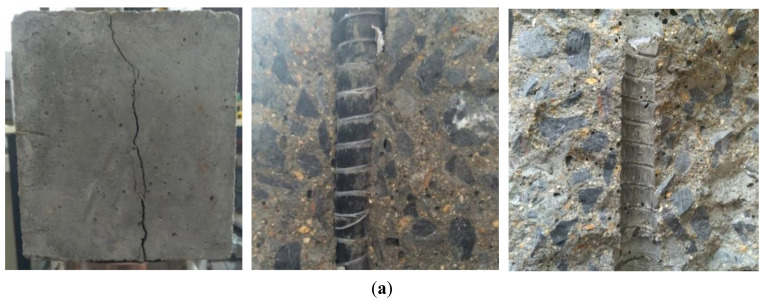

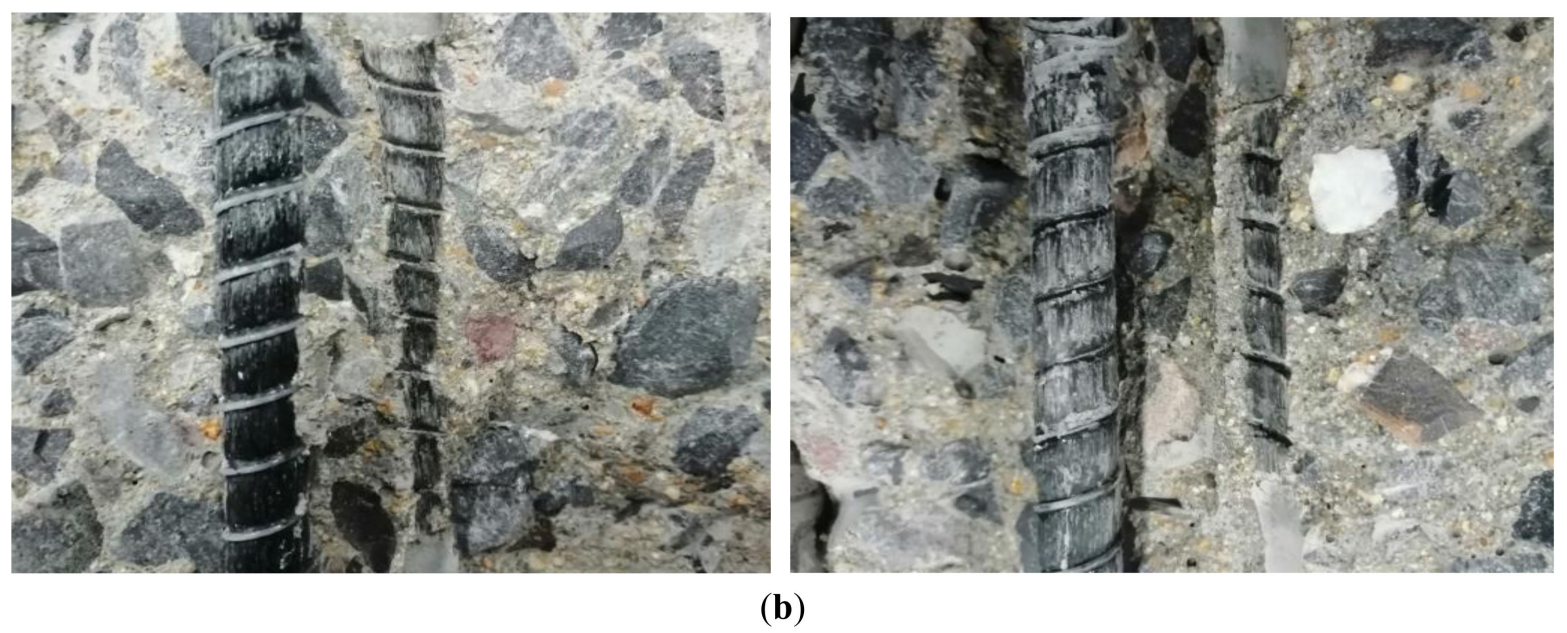
Photography of typical failure modes of glass fiber-reinforced polymer (GFRP) pull-out specimens: (**a**) splitting mode and (**b**) pull-out mode (Note: The pictures of pull-out mode were taken after the manual splitting of tested specimens) (see Table 3 Summary of test results of pull-out specimens).

The typical bond–slip (i.e., *τ*–*s*) curves of three types of reinforcements (i.e., FRP bars, steel strands, and steel bars) are summarized in Figure 5. Clearly, the *τ–s* curves in Figure 5a,d included three stages of ascending (from the initial point to point A), descending (from point A to point B), and residual (from point B to terminal point) sections, while the *τ–s* curve in Figure 5c only included two stages of ascending (from the initial point to point A), descending (from point A to point B) sections. In order to investigate the bond strength retention rate in the residual phase of FRP bar and steel bar specimens, the residual bond strength was defined as the maximum bond stress in the residual section of *τ–s* curve. Clearly, two characteristic points of the *τ–s* curve results were analyzed for FRP bars with pull-out failure mode (Figure 5a)—the first peak point (i.e., point A) of the *τ*–*s* curve corresponds to the maximum (or ultimate) bond stress *τ*_0_ and the slip *s*_0_ at the free end; the second peak point (i.e., point C) of the *τ*–*s* curve corresponds to the bond stress *τ*_1_ (i.e., residual bond strength) and the slip *s*_1_ at the free end. It is noted that, different from some previous studies in which point B in Figure 5a was taken as the residual extreme point and the bond stress at point B was analyzed, point C was considered as the residual extreme point in this study. This is because according to the definition of bond strength, the maximum value of stress in the *τ*–*s* curve is the bond strength, and thus the residual bond strength *τ*_1_ should be the maximum value of stress in the residual section. Similarly, for steel bar specimens, point C (residual bond strength point) and point B were considered to be coincident for steel bar in this study, as shown in Figure 5d. Moreover, for steel strand pull-out specimens since the residual section of the *τ–s* curve are missing in this study, the terminal point (i.e., point B) of descending section of the steel strand was approximated as the residual strength point (point C) for comparing with that of FRP and steel bars to analyze the bond strength retention rate, that is, point C and point B were considered to be coincident for steel strand in this study, as shown in Figure 5c. In addition, taking GFRP bars as an example, two kinds of typical failure modes of FRP pull-out specimens were shown in Figure 6.

In this study, the bond stress of pull-out specimens was taken as the average bond stress within the bond length of reinforcement. The equation for calculating the bond stress in between reinforcement and concrete is as follows [18]:

(1) $$\tau =\frac{P}{\pi dl}$$

where *τ* is the bond stress between reinforcement and concrete, *P* is the pull-out load, *d* is the diameter of the reinforcement, and *l* is the bond length of the reinforcement.

In addition, the bond strength (i.e., maximum bond stress) of reinforcement is indicated *τ*_0_, the residual bond strength (i.e., the maximum bond stress in residual section) is indicated as *τ*_1_, and the slips at the free end correspond to *τ*_0_ and *τ*_1_ were indicated as *s*_0_ and *s*_1_, respectively. It is noted that only the slip results at the free end were analyzed in this study.

All the experimental results of CFRP/GFRP/ steel strands and steel bars obtained in this study are summarized in Table 3 and Table 4, respectively. 

## 4. Discussions

### 4.1. Influence of Various Factors

This section investigates the effects of type of reinforcement, grade of concrete, bond length, diameter of reinforcement, and stirrup on the bond strength and bond-slip curves of four types of pull-out specimens [18].

#### 4.1.1. Effect of Type of Reinforcement

**Figure 7:**
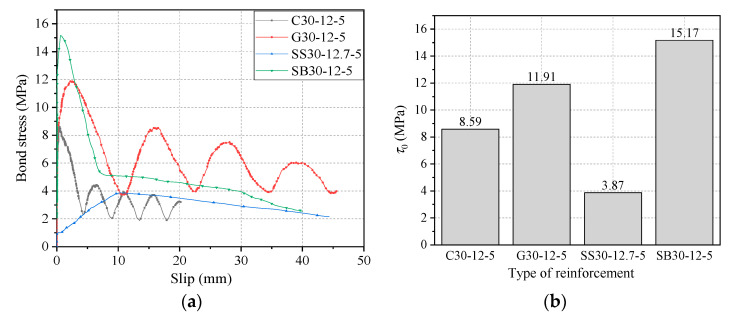
Comparison of four types of reinforcement materials in terms of (**a**) bond–slip curve and (**b**) bond strength.

As shown in Figure 7, the bond strength of CFRP bars is 56% of that of steel bars with the same diameter, bond length, and grade of concrete and the bond strength of GFRP bars is 3.32 MPa higher than that of CFRP bars. This is related to the surface morphology of the reinforcement. The surface of GFRP bars is rough compared to CFRP bars since the horizontal ribs were formed on the surface of GFRP bars through spirally winding bundles of glass fibers, which provide stronger mechanical interaction between the GFRP bar and concrete. The bond strength of steel strands is 25.5% of that of steel bars, and the gap between them is as high as 11.30 MPa. It can be derived that, among these four reinforcement materials, the bond strength of steel bars is the highest, followed by GFRP and CFRP bars, and the lowest is steel strand. It is also found that the residual bond strength of GFRP was increased by 4.12 MPa compared with CFRP bars. This was mainly due to the fact that the superficial glass fibers of GFRP bars were slightly broken during the pull-out process, while the concrete between the ribs was not damaged; therefore, there is still considerable friction between GFRP and concrete in the residual stage. Meanwhile, the value of *τ*_1_/*τ*_0_ is 0.72 and 0.56 for GFRP and CFRP bars, respectively. Since *τ*_1_/*τ*_0_ is the ratio of the residual bond strength to the bond strength, and the higher *τ*_1_/*τ*_0_ value indicates the higher bond strength retention rate, which means that the bond strength retention rate of GFRP bars is better than that of CFRP bars. From the bond–slip curve, it is found that the descending section of the steel strand was gentler compared with that of the steel bar, and the slip *s*_0_ at *τ*_0_ was much higher. Furthermore, the descending section of the steel strand was also almost equal to the residual section of the steel bar, and both curves coincide at a slip of 40 mm. This indicated that less loss of bond stress in the residual section and a higher bond strength retention rate of the steel strand than the steel bar. In addition, it can be found that the slope of ascending section of the *τ*–*s* curve of steel strand was much lower than that of CFRP, GFRP, and steel bars, which indicated the relatively lower bond stiffness between steel strand and concrete. This is the reason for the higher *s*_0_ of steel strand specimens than GFRP, CFRP, and steel bar specimens.

#### 4.1.2. Effect of Grade of Concrete

**Figure 8:**
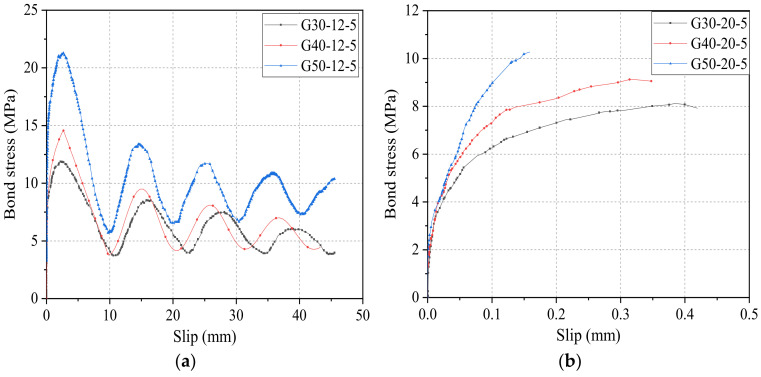
Bond–slip curves of GFRP bars with (**a**) diameter of 12 mm and (**b**) diameter of 20 mm.

Figure 8 shows the bond–slip curves of GFRP bars with diameters of 12 mm and 20 mm and bond lengths of 5*d* embedded in different grades of concrete. Clearly, for a concrete grade of C30, the bond strength of GFRP bars with a diameter of 12 mm and 20 mm is 11.91 MPa and 8.12 MPa, respectively, and the slip *s*_0_ is 2.73 mm and 0.38 mm, respectively. With the increase of grade of concrete from C30 to C40, the bond strength of GFRP bars with diameters of 12 mm and 20 mm increased by 2.7 MPa and 1 MPa (i.e., 22.7% and 12.3%), respectively. Moreover, the slip *s*_0_ was reduced by 0.06 mm and 0.07 mm (i.e., 2.2% and 18.4%), respectively. For concrete with a grade of C50, the bond strength of GFRP bars were 21.33 MPa and 10.27 MPa for 12 mm and 20 mm diameters, respectively, and the slip *s*_0_ were found to be 2.56 mm and 0.16 mm, respectively. This shows a further increase by 9.42 MPa and 2.15 MPa (i.e., 79.1% and 26.5%) in bond strength and surplus reduction by 0.17 mm and 0.22 mm (i.e., 6% and 57.9%) in *s*_0_, respectively. It can be concluded that, with the increase of grade of concrete, the bond strength of GFRP bars increases significantly, while the free-end slip *s*_0_ decreases to some extent. The outcomes of the remaining experimental groups were found to be similar and will not be repeated here.

*τ*_1_/*τ*_0_ is the ratio of residual bond strength to ultimate bond strength, which can reflect the degree of decay of the residual stage bond stress compared to the ultimate bond strength, and the higher its value indicates the higher bond strength retention rate. In the case of 12 mm diameter, in the concrete lightness grade C30, the residual bond strength is 8.57 MPa, and the ratio of residual bond strength to ultimate bond strength is 0.72. Similarly, for a concrete grade of C40, the residual bond strength is 9.50 MPa, and the ratio of residual bond strength to ultimate bond strength is 0.65. Compared with the concrete grade of C30, the residual bond strength increases by 0.93 and bond strength retention rate decreases by 0.07 for a concrete grade of C40. Furthermore, for a concrete grade of C50, the residual bond strength is 13.53 MPa and bond strength retention rate is 0.63, and the residual bond strength increases by 4.96 and bond strength retention rate decreases by 0.09 compared with the concrete grade of C30. It is found that the bond strength of the residual section gradually increases as the concrete strength increases. However, the value of *τ*_1_/*τ*_0_ keeps decreasing, which indicates that although increasing the strength of concrete can increase the ultimate bond strength and residual bond strength of GFRP, it can also reduce the bond strength retention rate of GFRP bars.

**Figure 9:**
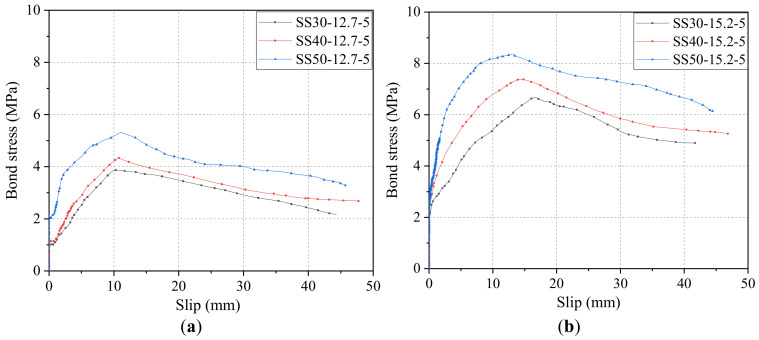
Bond–slip curves of steel strand with (**a**) diameter of 12.7 mm and (**b**) diameter of 15.2 mm.

Figure 9 shows the effect of concrete grade on the bonding performance of the steel strand. Figure 9a shows the bond–slip curves of the steel strand with a diameter of 12.7 mm and a bond length of five times the given diameter at different concrete strength levels. From Figure 9a, it can be derived that, when the concrete design strength becomes C30, the ultimate bond strength of the strand was 3.87 MPa, and the free-end slip at the limit point was 10.31 mm. In addition, when the concrete design strength reaches C40, the bond strength of the steel strand was found to be 4.34 MPa, and the free-end slip at the limit point was 10.79 mm. Compared with the concrete grade of C30, the bond strength of the steel strand increased by 0.47 MPa, which shows an increment of 12.1%, and the free-end slip at the limit point, which also shows an increment by 0.48 mm, an increase of 4.7. Furthermore, for the concrete grade of C50, the bond strength of the steel strand was 5.32 MPa, and the free-end slip at the limit point was 11.07 mm; compared with the concrete grade of C30, the bond strength of the steel strand showed an increment of 1.45 MPa, an increase of 37.5%, and the free-end slip at the limit point showed an increment of 0.76 mm, an increase of 7.4%. It can be concluded that the bond strength of the strand gradually increases with the increase of concrete strength.

Figure 9b shows the diameter of 15.2 mm and the bond length of five times the given diameter of the steel strand at different concrete strength levels. From the figure, when the concrete design strength becomes C30, the ultimate bond strength of the steel strand was 6.68 MPa, the limit point free-end slip of the steel strand was 16.71 mm. In addition, when the concrete design strength reaches C40, the bond strength of the steel strand was 7.37 MPa, and the limit point free-end slip of the steel strand was 14.33 mm. Compared with the concrete grade of C30, the bond strength of the steel strand increased by 0.69 MPa, which shows an increment of 10.3%, and the limit points free-end slip decreased by 2.38 mm, which shows a reduction of 14.2. Furthermore, for the concrete grade of C50, the bond strength of the steel strand was 8.34 MPa, and the limit point free-end slip of the steel strand was 13.17 mm; compared with the concrete grade of C30, the bond strength of the steel strand showed an increment of 1.66 MPa, an increase of 24.9%, and the limit point free-end slip decreased by 35.4 mm, which showed a reduction of 21.2%. The findings of the rest of the experimental groups were similar and will not be repeated.

It can be concluded that, with the increase in the strength of the substrate concrete, the bond strength of the steel strand increases, and the change in the free-end slip of the limit point were not consistent for different diameters of the strand; for the 12.7 mm diameter steel strand, with the increase in the concrete strength, the free-end slip of the limit point increases, while for the 15.2 mm diameter strand, with the increase in the concrete strength, the free-end slip of the limit point decreases. The free-end slippage decreases as the concrete strength increases.

#### 4.1.3. Effect of Bond Length

**Figure 10:**
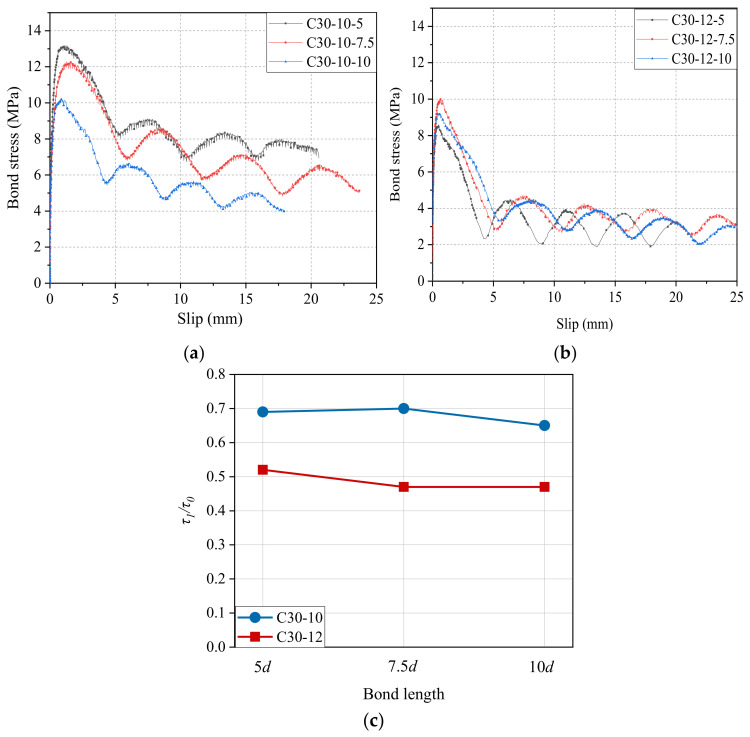
Bond–slip curves and *τ*_1_*/τ*_0_ results of CFRP bars with different bond lengths: (**a**) diameter of 10 mm; (**b**) diameter of 12 mm; and (**c**) comparison of *τ*_1_*/τ*_0_.

Figure 10a,b shows the effect of bond length on the bond–slip curves of CFRP bars with diameters of 10 mm and 12 mm to the concrete grade of C30. Figure 10c further compares *τ*_1_/*τ*_0_ values of CFRP bars with different bond lengths in both diameters. It can be seen that as the bond length of the CFRP bar increases, *τ*_1_/*τ*_0_ tends to decrease, indicating the decreased bond strength retention rate in the residual phase.

**Figure 11:**
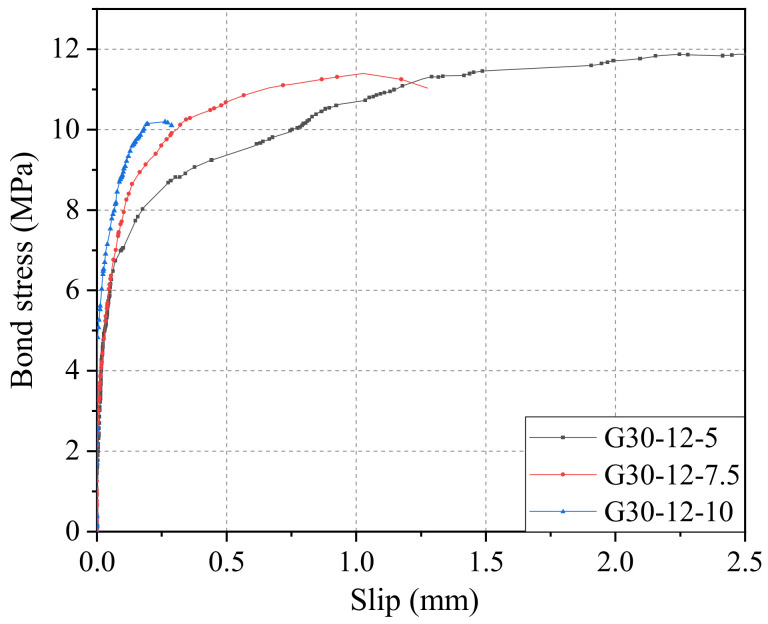
Bond–slip curves of GFRP bars with different bond lengths.

The effect of bond length on the bond–slip curves of the GFRP bar with a diameter of 12 mm to the concrete grade of C30 is shown in Figure 11. It is found that the bond strength *τ*_0_ and the related slip *s*_0_ of GFRP bars with a diameter of 12 mm were 11.91 MPa and 2.73 mm for the bond length of 5*d*, 11.40 MPa and 1.03 mm for the bond length of 7.5*d*, and 10.19 MPa and 0.26 mm for the bond length of 10*d*, respectively. Clearly, as the increase of bond length from 5*d* to 7.5*d* and then to 10*d*, the bond strength value started to decline by 0.51 MPa and 1.21 MPa (i.e., 4.2% and 10.6%), while it also found a dramatic reduction of 1.70 mm and 0.77 mm (i.e., 62.3% and 74.8%) in the slip *s*_0_. A similar change trend of *τ*_0_ and *s*_0_ with bond length can also be found for GFRP bars with a diameter of 20 mm as (seen in Table 3), and repeated discussion will not be forwarded here.

**Figure 12:**
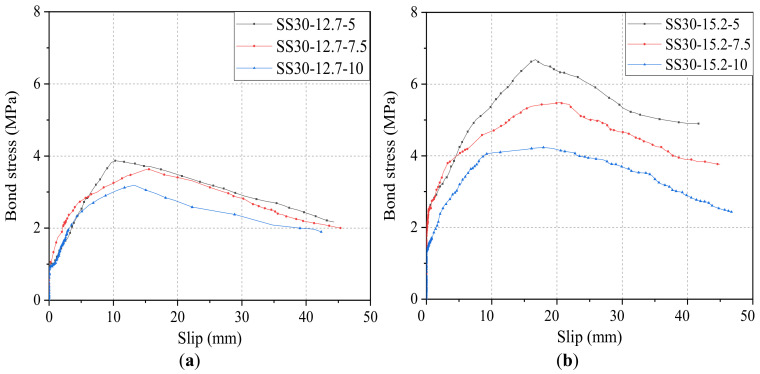
Bond–slip curves of steel strands with different bond lengths: (**a**) diameter of 12.7 mm and (**b**) diameter of 15.2 mm.

Figure 12 shows the effect of bond length on bond–slip curves of steel strands with diameters of 12.7 mm and 15.2 mm to the concrete grade of C30. As shown in Figure 12a, for steel strands pull-out specimens with a diameter of 12.7 mm and concrete grade of C30, its *τ*_0_ and *s*_0_ were 3.87 MPa and 10.11 mm for the bond length of 5*d*, 3.63 MPa and 15.56 mm for the bond length of 7.5*d*, and 3.19 MPa and 13.25 mm for the bond length of 10*d*, respectively. It is clear that with the increase of bond length from 5*d* to 7.5*d*, and then to 10*d*, the bond strength starts to decline by 0.24 MPa and 0.44 MPa (i.e., 6.2% and 12.1%). Similarly, as shown in Figure 12b, for steel strands pull-out specimens with a diameter of 12.7 mm and concrete grade of C30, its *τ*_0_ and *s*_0_ were 6.68 MPa and 16.71 mm for the bond length of 5*d*, 5.49 MPa, and 20.57 mm for the bond length of 7.5*d*, and 4.23 MPa and 17.91 mm for the bond length of 10*d*, respectively. There was a reduction in bond strength by 1.19 MPa and 1.26 MPa (i.e., 17.8% and 23.0%). Therefore, it can be concluded that, in the case of the same grade of concrete and diameter of steel strands, as the bond strength increases, the bond strength *τ*_0_ gradually decreases, but the variation trend of related slip *s*_0_ is irregular.

#### 4.1.4. Effect of Diameter of Reinforcement

**Figure 13:**
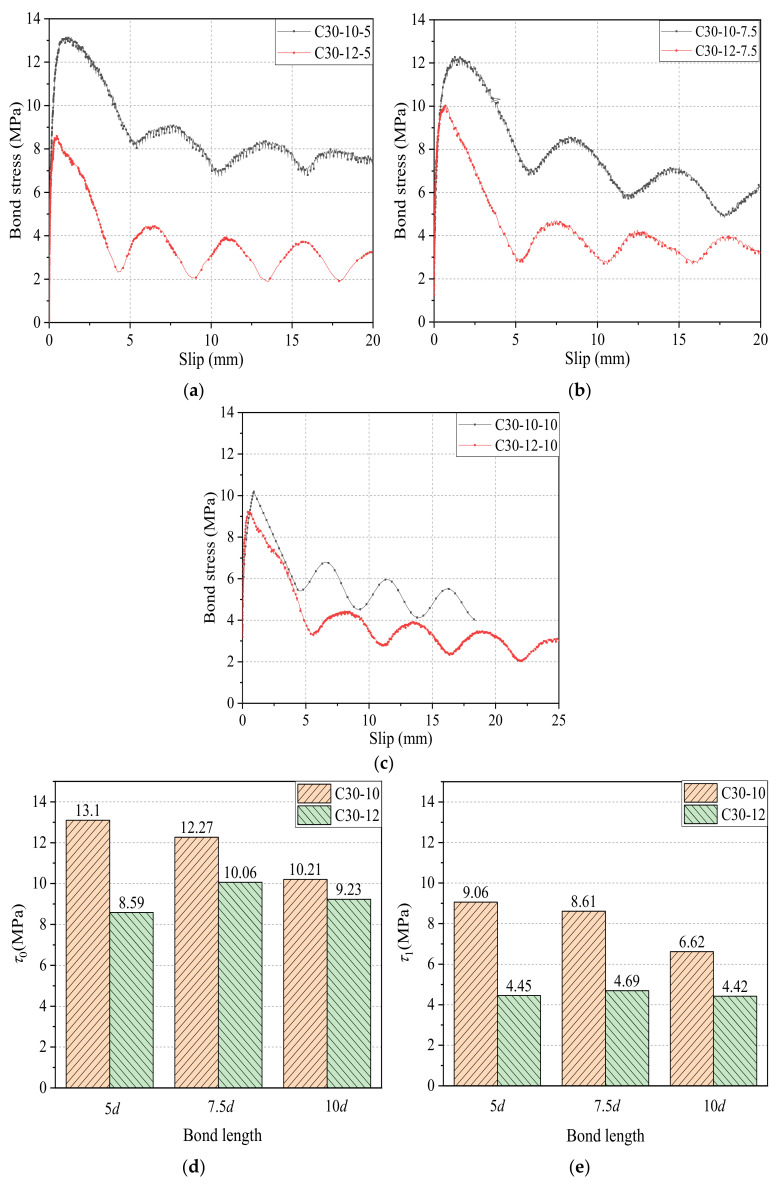
Bond–slip curves and *τ*_0_ and *τ*_1_ results of CFRP bars with different diameters: (**a**) bond length of 5*d*; (**b**) bond length of 7.5*d*; (**c**) bond length of 10*d*; (**d**) comparison of bond strength *τ*_0_; and (**e**) comparison of residual bond strength *τ*_1_.

Figure 13a–c shows the effect of diameter on bond–slip curves of CFRP bars pull-out specimens with three kinds of bond lengths (5*d*, 7.5*d*, and 10*d*) and concrete grade of C30, and Figure 13c,d further summarized *τ*_0_ and *τ*_1_ results. As shown in Figure 13, the *τ*_0_ values of CFRP bars with diameters of 10 mm and 12 mm were13.11 MPa and 8.59 MPa for the bond length of 5*d*, 12.27 MPa, and 10.06 MPa for the bond of 7.5*d*, and 10.21 MPa and 9.23 MPa for the bond of 10*d*, respectively. The *τ*_1_ values of CFRP bars with a diameter of 10 mm and 12 mm are 9.06 MPa and 4.45 MPa for the bond of 5*d*, 8.61 MPa and 4.69 MPa for the bond of 7.5d, and 6.62 MPa and 4.42 MPa for the bond length of 10*d*, respectively. It is obvious that compared with 10 mm-diameter CFRP bars, the *τ*_0_ of 12-mm diameter CFRP bars decreases by 4.52 MPa, 2.21 MPa, and 0.98 MPa (i.e., 34.5%, 18.0%, and 9.6%) for the bond length of 5*d*, 7.5*d*, and 10*d*, respectively. Similarly, compared with CFRP bars with a diameter of 10 mm, the *τ*_1_ of CFRP bars with a diameter of 12 mm decreases by 4.61 MPa, 3.92 MPa, and 2.20 MPa (i.e., 50.9%, 45.5%, and 33.2%) for the bond length of 5*d*, 7.5*d*, and 10*d*, respectively. It can be concluded that as the diameter increases, both the bond strength *τ*_0_ and the residual bond strength *τ*_1_ decrease. This is mainly because the larger diameter causes the smaller relative bond area, the lower relative rib height, more voids at the bond interface zone between CFRP bars and concrete, and the presence of the effect of shear hysteresis [40]. In addition, Figure 13 also shows that the ratio of *τ*_1_*/τ*_0_ decreases, and the bond strength retention rate of GFRP bars start to decline as the diameter of CFRP bars increases. To avoid repetition of figures, the detailed comparison results of *τ*_1_*/τ*_0_ refers to Figure 10c.

**Figure 14:**
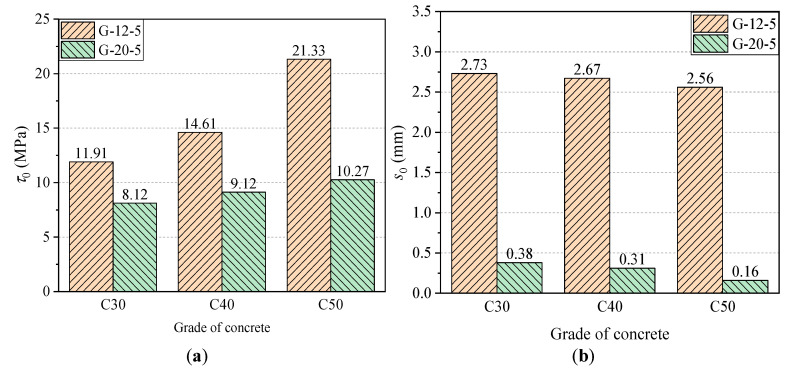
The effect of diameter on *τ*_0_ and *s*_0_ results of GFRP bars: (**a**) comparison of bond strength *τ*_0_ and (**b**) comparison of *s*_0_.

Figure 14 shows a comparison of *τ*_0_ and *s*_0_ of GFRP bars with 12 mm and 20 mm diameters for the bond length of 5*d* and the same grade of concrete. As shown in Figure 14a, the *τ*_0_ values of CFRP bars with diameters of 10 mm and 12 mm were 11.91 MPa and 8.12 MPa for C30 concrete, 14.61 MPa and 9.12 MPa for C40 concrete, and 21.33 MPa and 10.27 MPa for C50 concrete, respectively. It is apparent that compared with 10mm-diameter GFRP bars, the *τ*_0_ of 12 mm-diameter GFRP bars decreases by 3.79 MPa, 5.49 MPa, and 11.06 MPa (i.e., 31.8%, 37.6%, and 51.9%) for the bond length of 5*d*, 7.5*d*, and 10*d*, respectively. Similarly, as shown in Figure 14b, the *s*_0_ values of CFRP bars with diameters of 10 mm and 12 mm were 2.73 mm and 0.38 mm for C30, 2.67 mm and 0.31 mm for C40 concrete, and 2.56 mm and 0.16 mm for C50 concrete, respectively. Furthermore, compared with 10 mm-diameter GFRP bars, the *s*_0_ of 12mm-diameter GFRP bars decreases sharply by 2.35 mm, 2.36 mm, and 2.40 mm (i.e., 86.1%, 88.4%, and 94.8%) for the bond length of 5*d*, 7.5*d*, and 10*d*, respectively. The above results show that, in the case of the same bond length, the larger diameter of GFRP bars causes the lower bond strength *τ*_0_ and *s*_0_, and the effect of diameter was enhanced as increasing the concrete strength.

**Figure 15:**
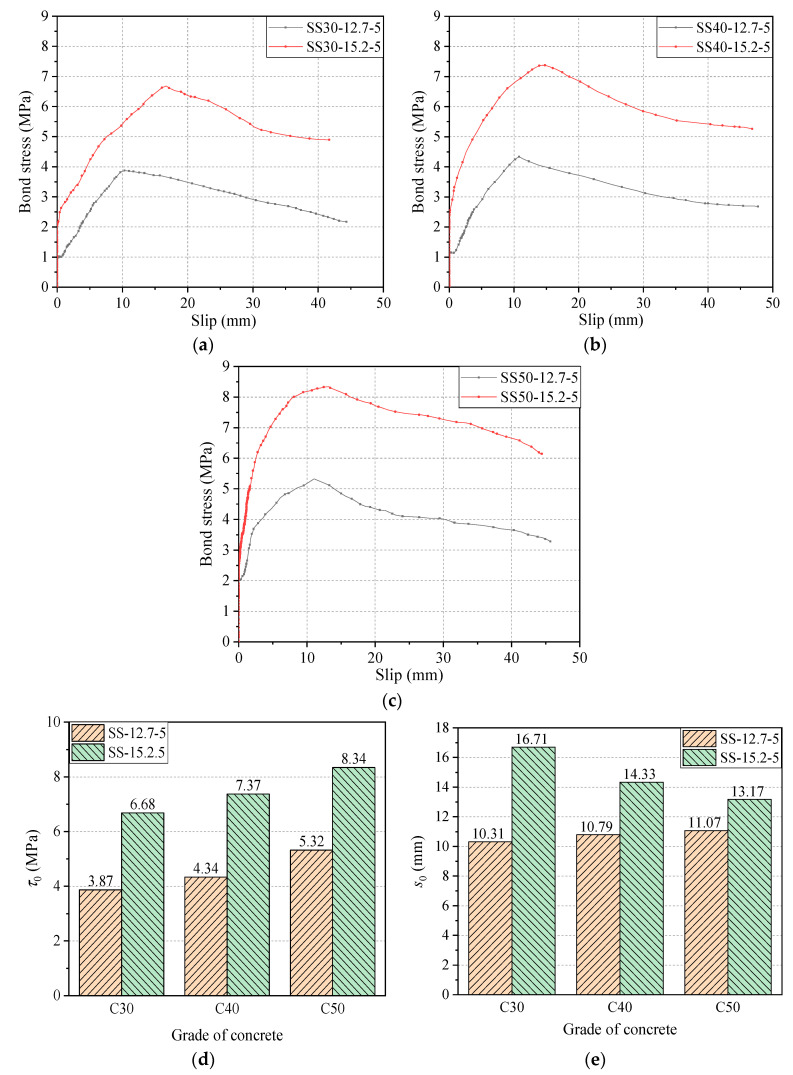
Bond–slip curves and *τ*_0_ and *s*_0_ steel strands with different diameters: (**a**) concrete of grade of C30; (**b**) concrete of grade of C40; (**c**) concrete of grade of C50; (**d**) comparison of bond strength *τ*_0_; and (**e**) comparison of *s*_0_.

Figure 15a–c shows the effect of diameter on bond–slip curves of steel strands pull-out specimens with three grades of concrete (C30, C40, and C50) and bond length of 5*d*. Figure 15c,d further summarized *τ*_0_ and *s*_0_ results. As shown in Figure 15, the *τ*_0_ values of steel strands with diameters of 12.7 mm and 15.2 mm are 3.87 MPa and 6.68 MPa for C30 concrete, 4.34 MPa and 7.37 MPa for C40 concrete, and 5.32 MPa and 8.34 MPa for C50 concrete, respectively. The *τ*_1_ values of steel strands with a diameter of 12.7 mm and 15.2 mm are 10.31 MPa and 16.71 MPa for C30 concrete, 10.79 MPa and 14.33 MPa for C40 concrete, and 11.07 MPa and 13.17 MPa for C50 concrete, respectively. Clearly, compared with 12.7 mm-diameter steel strands, the *τ*_0_ of 15.2 mm-diameter steel strands increases by 2.81 MPa, 3.03 MPa, and 3.02 MPa (i.e., 72.6%, 69.8%, and 56.8%) for C30, C40, and C50 concrete, respectively. Similarly, compared with 12.7 mm-diameter steel strands, the *τ*_1_ of 15.2 mm-diameter steel strands increases by 6.40 MPa, 3.54 MPa, and 2.10 MPa (i.e., 62.1%, 32.8%, and 19.0%) for C30, C40, and C50 concrete, respectively. It can be concluded that, in the case of the same bond length, the larger diameter of steel strands causes the higher bond strength *τ*_0_ and *s*_0_ of steel strands with concrete. This can be explained by the fact that the thicker steel strands result in the greater contact area with concrete, and likewise, the larger gap formed by spiral winding of steel strands resulted in the greater mechanical interaction between concrete and ribs of steel strands. Meanwhile, with the increase of grade of concrete, the effect of diameter on bond strength of steel strands was offset by the effect of the grade of concrete, which has been discussed in Section 4.1.2.

#### 4.1.5. Effect of Stirrup

**Figure 16:**
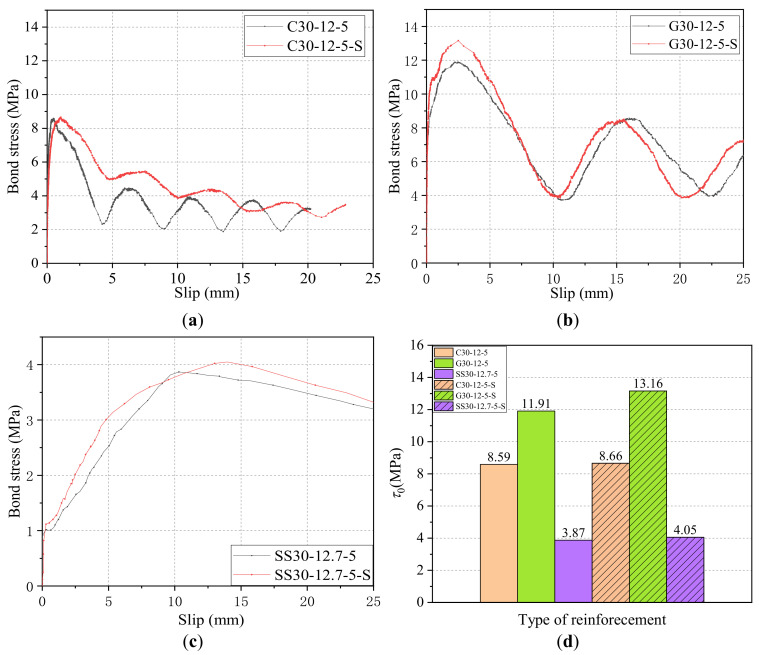
Bond–slip curves and *τ*_0_ results of reinforcements with/without stirrups: (**a**) CFRP bars; (**b**) GFRP bars; (**c**) steel strands; and (**d**) comparison of *τ*_0_.

Figure 16a–c shows the effect of stirrups on the bond–slip curves of CFRP, GFRP bars, and steel strands pull-out specimens with a diameter of 12 mm (or 12.7 mm) with a concrete grade of C30 and bond length of 5*d*. Figure 16d further summarized the *τ*_0_ results. As shown in Figure 16a, the test results of CFRP bars without and with stirrups are 8.59 MPa and 8.66 MPa for *τ*_0_, 0.47 mm and 1.06 mm for *s*_0_, 4.45 MPa and 5.48 MPa for *τ*_1_, and 5.94 mm and 7.16 mm for *s*_1_, respectively. Clearly, compared with CFRP bars without stirrups, the *τ*_0_, *s*_0_, *τ*_1_, and *s*_1_ of CFRP with stirrups increases by 0.07 MPa, 0.59 mm, 1.03 MPa and 1.22 mm (i.e., 0.8%, 125.5%, 23.1%, and 20.5%), respectively. As shown in Figure 16b, the test results of GFRP bars without and with stirrups are 11.91 MPa and 13.16 MPa for *τ*_0_, 2.73 mm, and 2.70 mm for *s*_0_, 8.57 MPa and 8.60 MPa for *τ*_1_, and 15.57 mm and 15.53 mm for *s*_1_, respectively. Clearly, compared with CFRP bars without stirrups, the *τ*_0_ and *τ*_1_ of CFRP with stirrups increase by 1.25 MPa and 0.03 MPa (i.e., 10.5% and 0.4%), respectively, while the *s*_0_ and *s*_1_ of CFRP with stirrups increase slightly by 0.03 mm and 0.04 mm (i.e., 1% and 0.3%), respectively. As shown in Figure 16c, the *τ*_0_ of steel strands without and with stirrups are 3.97 MPa and 4.05 MPa for *τ*_0_, and 10.31 mm and 13.97 mm for *s*_0_, respectively. Clearly, compared with steel strands without stirrups, the *τ*_0_ and *s*_0_ of steel strands with stirrups increase by 0.08 MPa and 3.66 mm (i.e., 2.0% and 35.5%), respectively. In addition, the results of the steel bars groups without and with stirrups were similar and will not be repeated here.

It can be concluded that, for the CFRP, GFRP, and steel strands, adding stirrups can increase the bond strength and the slip *s*_1_. This was found to be because the stirrup has a restraining effect on the circumferential deformation of the surrounding concrete of reinforcement, which can delay the development of cracks and reduce the damage of the concrete between the surficial ribs on the reinforcement. Meanwhile, the stirrups can increase the oblique squeezing force of the concrete on the cross ribs, which increases the mechanical interaction force between reinforcement and concrete to a certain extent and finally increase the bond strength.

### 4.2. Discussion of Bond Strength Retention Rate of GFRP/CFRP Bars and Steel Strands

Finally, the bond strength retention rate of three types of reinforcements (CFRP, GFRP bars, and strands) was analyzed and compared in this section.

**Figure 17:**
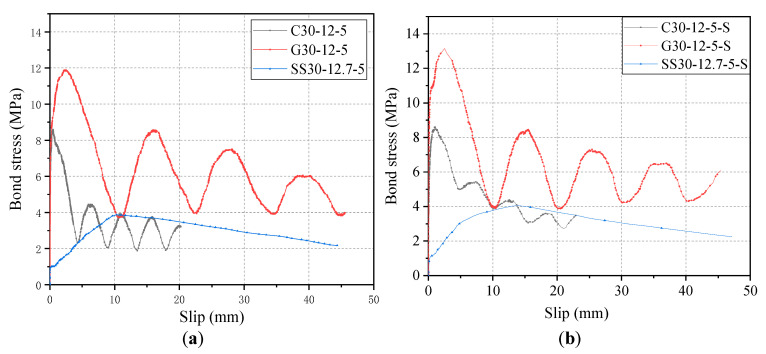
Comparison of bond–slip curves of CFRP/GFRP bars and steel strands: (**a**) specimens without stirrup and (**b**) specimens with the stirrup.

The bond–slip curves of the CFRP bar, GFRP bar, and steel strand are compared in Figure 17. It can be seen from Figure 17 that the residual segment of the bond–slip curve of CFRP and GFRP bars were gradually decaying wave-like distribution, while the bond-slip curve of steel strands as in a straight-line downward trend without a residual segment after the peak point.

As shown in Figure 17a, the residual bond strength of G30-12-5 and C30-12-5, and SS-30-12.7-5 were 8.57 MPa, 4.45 MPa, and 2.10 MPa, respectively. It is noted that the residual bond strength of specimen SS30-12.7-5 was found to be the bond stress at the slip of 46.87 mm. Clearly, the residual bond strength of the CFRP bar and steel strand without stirrup were 72.1% and 24.5% of that of the GFRP bar with the stirrup. As shown in Figure 17b, the residual bond strength of G30-12-5-S, C30-12-5-S, and SS-30-12.7-5-S were 8.60 MPa, 5.48 MPa, and 2.21 MPa, respectively. The residual bond strength of specimen SS30-12.7-5-S was found to be the bond stress at the slip of 47.23 mm. Clearly, the residual bond strength of CFRP bar and steel strand with stirrup were 63.7% and 25.7% of that of GFRP bar with the stirrup, respectively. In addition, comparing the key evaluation index of bond strength retention rate (*τ*_1_/*τ*_0_) of three reinforcements in both cases of Figure 17, the *τ*_1_/*τ*_0_ values of GFRP and CFRP bars are 0.65–0.72 and 0.52–0.63, respectively, while the *τ*_1_/*τ*_0_ values for steel strand are 0.53–0.55. Meanwhile, the *τ*_1_/*τ*_0_ values for steel bars are as low as 0.33–0.39. Since the higher *τ*_1_/*τ*_0_ suggests the higher bond strength retention rate, it can be concluded that the bond strength retention rate starts to decrease from left to right (GFRP, CFRP, steel strand, and steel bar), respectively.

## 5. Conclusions

In this paper, the bond–slip tests were conducted between three different high-strength reinforcement materials, CFRP bars, GFRP bars, and steel strands, and the effect of reinforcement type, reinforcement diameter, concrete strength grade, bond length, and stirrup on the failure were analyzed. Further, bond strength, residual bond strength, slip at the free end and bond strength retention rate, were also investigated. The bond–slip curves of different reinforcement materials were compared and analyzed. In particular, the following conclusions can be drawn from this study:

(1) Under the same conditions, the bond strength of CFRP, GFRP bars, and steel strands are 55%, 78%, and 25% of steel bars, respectively;

(2) Under the same conditions, the residual bond strength of GFRP bars is greater than that of CFRP bars, and the bond stress in the residual section is similar to that of CFRP bars;

(3) The bond strength retention rate of the GFRP bar is higher than that of CFRP bars and steel strands, whereas the steel bar has the lowest bond strength retention rate in this study;

(4) For CFRP, the larger the bond length and the larger the diameter of the bar, the residual bond strength and bond strength retention rate start to decline. Moreover, adding stirrup can also increase the residual bond strength of the CFRP bar to a small extent;

(5) For GFRP, with the increase of the grade of concrete, the bond strength and residual bond strength increase, but the bond strength retention rate decreases. Increasing the GFRP bar diameter and bond length will result in bond strength reduction. The addition of stirrups can increase the bond strength of the GFRP bar to a small extent;

(6) For the steel strand, the bond strength increases with the increase of grade of concrete. Furthermore, with the increase in bond length, the bond strength declines; the larger the diameter of the steel strand is, the greater the bond strength, which is against the above CFRP and GFRP bars. The addition of stirrups can also increase the bond strength of the steel strand to a small extent.

## Figures and Tables

**Table 1:** Mechanical properties of high-strength reinforcement materials.

Bar Type	Tensile Strength (MPa)	Modulus of Elasticity (GPa)
CFRP-12 mm	2322	144
CFRP-20 mm	2108	143
GFRP-12 mm	991	44
GFRP-20 mm	744	45
Steel strand-12.7 mm	1860	195
Steel strand-15.2 mm	1860	195

**Table 2:** Mix proportion and compressive strength of concrete.

Design Grade	W/C	Water (kg/m^3^)	Cement (kg/m^3^)	River Sand (kg/m^3^)	Gravel (kg/m^3^)	Water Reducer (kg/m^3^)	Compressive Strength (MPa)
C30	0.62	215	347	701	1137	0	39.3
C40	0.48	214	448	614	1124	0	46.9
C50	0.40	149	375	760	1115	1.49	60.7

Note: W/C represents the water to cement ratio.

**Table 3:** Summary of test results of carbon fiber-reinforced polymer (CFRP) and GFRP bars.

Specimen	Diameter *d* (mm)	Bond Length *l* (mm)	Bond Strength *τ*_0_ (MPa)	Residual Bond Strength *τ*_1_ (MPa)	*τ*_1_/*τ*_0_	*s*_0_ (mm)	*s*_1_ (mm)	*s*_1_/*s*_0_	Failure Mode
C30-10-5	10	5*d*	13.11 ± 1.21	9.06 ± 0.24	0.69	1.02 ± 0.02	7.68 ± 1.02	7.53	Pull-out
C30-10-7.5	10	7.5*d*	12.27 ± 2.98	8.61 ± 2.42	0.70	1.27 ± 0.12	8.24 ± 1.51	6.49	Pull-out
C30-10-10	10	10*d*	10.21 ± 2.41	6.62 ± 1.72	0.65	0.88 ± 0.01	5.84 ± 0.28	6.63	Pull-out
C30-12-5	12	5*d*	8.59 ± 0.39	4.45 ± 0.05	0.52	0.47 ± 0.05	5.94 ± 1.38	12.64	Pull-out
C30-12-7.5	12	7.5*d*	10.06 ± 1.65	4.69 ± 1.82	0.47	0.70 ± 0.61	7.51 ± 2.91	10.72	Pull-out
C30-12-10	12	10*d*	9.23 ± 1.76	4.42 ± 1.01	0.47	0.56 ± 0.08	7.85 ± 0.67	14.02	Pull-out
C30-12-5-S	12	5*d*	8.66 ± 1.11	5.48 ± 1.44	0.63	1.06 ± 0.25	7.16 ± 4.93	6.75	Pull-out
G30-12-5	12	5*d*	11.91 ± 1.13	8.57 ± 0.67	0.72	2.73 ± 0.05	15.57 ± 0.48	5.70	Pull-out
G30-12-7.5	12	7.5*d*	11.40 ± 1.02	-	-	1.03 ± 0.84	-	-	Splitting
G30-12-10	12	10*d*	10.19 ± 0.92	-	-	0.26 ± 0.07	-	-	Splitting
G30-20-5	20	5*d*	8.12 ± 0.50	-	-	0.38 ± 0.11	-	-	Splitting
G30-20-7.5	20	7.5*d*	7.91 ± 0.04	-	-	0.13 ± 0.02	-	-	Splitting
G40-12-5	12	5*d*	14.61 ± 1.11	9.50 ± 0.69	0.65	2.67 ± 0.37	14.39 ± 0.44	5.39	Pull-out
G40-20-5	20	5*d*	9.12 ± 0.68	-	-	0.31 ± 0.03	-	-	Splitting
G50-12-5	12	5*d*	21.33 ± 1.02	13.53 ± 0.51	0.63	2.56 ± 0.14	14.61 ± 0.11	5.71	Pull-out
G50-20-5	20	5*d*	10.27 ± 1.29	-	-	0.16 ± 0.02	-	-	Splitting
G30-12-5-S	12	5*d*	13.16 ± 1.15	8.60 ± 0.18	0.65	2.70 ± 1.07	15.53 ± 0.42	5.75	Pull-out

Note: The actual compressive strength of C30 concrete for CFRP specimens was measured as 36.7 MPa; the compressive strengths of concrete for the rest specimens refer to the data listed in Table 1; “-” represents “not available”.

**Table 4:** Summary of test results of steel strands and steel bars.

Specimen	Diameter *d* (mm)	Bond Length *l* (mm)	Bond Strength *τ*_0_ (MPa)	Residual Bond Strength *τ*_1_ (MPa)	*τ*_1_/*τ*_0_	*s*_0_ (mm)	*s*_1_ (mm)	*s*_1_/*s*_0_	Failure Mode
SS30-12.7-5	12.7	5*d*	3.87 ± 0.71	2.10 ± 0.80	0.53	10.31 ± 4.18	46.87 ± 0.85	4.55	Pull-out
SS30-12.7-7.5	12.7	7.5*d*	3.63 ± 1.18	2.00 ± 0.29	0.55	15.56 ± 2.48	43.89 ± 4.33	2.82	Pull-out
SS30-12.7-10	12.7	10*d*	3.19 ± 2.49	1.93 ± 0.05	0.61	13.25 ± 2.55	46.13 ± 0.17	3.48	Pull-out
SS30-15.2-5	15.2	5*d*	6.68 ± 1.01	4.48 ± 0.78	0.67	16.71 ± 2.06	44.99 ± 0.52	2.69	Pull-out
SS30-15.2-7.5	15.2	7.5*d*	5.49 ± 0.82	3.73 ± 0.68	0.68	20.57 ± 2.12	46.52 ± 0.57	2.26	Pull-out
SS30-15.2-10	15.2	10*d*	4.23 ± 0.46	2.91 ± 0.42	0.69	17.91 ± 5.62	44.75 ± 0.18	2.50	Pull-out
SS40-12.7-5	12.7	5*d*	4.34 ± 0.49	2.71 ± 0.19	0.62	10.79 ± 4.54	45.19 ± 0.23	4.19	Pull-out
SS40-15.2-5	15.2	5*d*	7.37 ± 1.43	5.23 ± 1.71	0.71	14.33 ± 1.65	45.99 ± 0.63	3.21	Pull-out
SS50-12.7-5	12.7	5*d*	5.32 ± 0.86	3.44 ± 0.55	0.65	11.07 ± 7.26	46.98 ± 0.35	4.24	Pull-out
SS50-15.2-5	15.2	5*d*	8.34 ± 0.69	6.15 ± 0.63	0.74	13.17 ± 1.09	45.59 ± 0.41	3.46	Pull-out
SS30-12.7-5-S	12.7	5*d*	4.05 ± 0.14	2.21 ± 0.55	0.55	13.97 ± 0.88	47.23 ± 0.28	3.38	Pull-out
SB30-12-5	12	5*d*	15.17 ± 0.69	5.08 ± 0.18	0.33	0.60 ± 0.13	8.08 ± 0.57	13.47	Pull-out
SB30-12-5-S	12	5*d*	15.72 ± 0.64	6.19 ± 0.49	0.39	1.27 ± 0.25	7.96 ± 0.37	6.27	Pull-out

## Data Availability

The raw/processed data required to reproduce these findings cannot be shared at this time as the data also forms part of an ongoing study.
